# TARDBP Mediates the MAP3K11/SLC3A2/GPX4 Axis in Alzheimer's Disease Rats by Enhancing KRAS mRNA Stability

**DOI:** 10.1111/jcmm.71181

**Published:** 2026-05-17

**Authors:** QiTao Zhao, YaDong Yu, FuJiang Wang, Ya Wang, Peng Shao, LianDong Zhao

**Affiliations:** ^1^ Department of Neurology Xuzhou Medical University Affiliated Hospital of Huaian Huai'an Jiangsu China; ^2^ Department of Neurology Lianshui County People's Hospital Huai'an Jiangsu China; ^3^ Department of Neurological Medicine Siyang Hospital of Traditional Chinese Medicine Suqian Jiangsu China; ^4^ Department of Physiology Nanjing Medical University Nanjing Jiangsu China

**Keywords:** Alzheimer's disease, ferroptosis, KRAS, MAP3K11/SLC3A2/GPX4 pathway, TARDBP

## Abstract

Ferroptosis is an emerging pathological mechanism in Alzheimer's disease (AD). The aim of the present study was to investigate the potential mechanisms by which TARDBP is involved in AD by promoting ferroptosis. An AD rat model was established by injecting homocysteine (Hcy). Memory function was assessed using the Morris water maze test and contextual fear conditioning test. Hippocampal neurons' morphology was observed by HE staining, and intracellular iron deposition in the hippocampus was evaluated by Perls' blue staining. PC12 cells were treated with 20 μM Aβ_1–42_ to establish an AD cell model in vitro. Cell viability was measured by MTT assay; LDH release, intracellular ROS levels and Fe^2+^ concentrations were determined. The mRNA stability of KRAS was assessed by actinomycin D assay. Activation of the MAP3K11/SLC3A2/GPX4 pathway was assessed by Western blot. Treatment with Fer‐1 or down‐regulation of TARDBP improved memory function and reduced intracellular iron deposition in the hippocampus of AD rats. Furthermore, these interventions inhibited Aβ_1–42_‐induced PC12 cell damage, ROS production and iron accumulation. Mechanistically, down‐regulating TARDBP reduced the mRNA stability of KRAS, inhibited MAP3K11 expression and subsequently promoted the expression of SLC3A2 and GPX4. Conversely, up‐regulation of KRAS reversed the protective effects induced by TARDBP knockdown in both AD rats and Aβ_1–42_‐induced PC12 cells. TARDBP promotes the development of AD by enhancing the mRNA stability of KRAS, thereby mediating the MAP3K11/SLC3A2/GPX4 axis to induce ferroptosis.

## 1 Introduction

It is estimated that Alzheimer's disease (AD) accounts for 60%–80% of all dementia cases [1]. In AD, amyloid‐β (Aβ) is deposited extracellularly as diffuse and neuritic plaques, while hyperphosphorylated tau tangles are present intracellularly as neurofibrillary tangles [2]. As complex neurochemical and genetic factors are involved in AD pathogenesis, the exact mechanism remains unclear [3].

Neuronal ferroptosis, an iron‐dependent form of programmed cell death characterized by the excessive production of lipid peroxides, has been linked to AD pathology [4, 5, 6, 7, 8, 9]. An increased iron level in the hippocampus, cortical lobe and basal ganglia correlates with the accumulation of Aβ plaques [10, 11]. Additionally, hallmarks of ferroptosis, including disruptions in iron metabolism, glutamate excitotoxicity and lipid reactive oxygen species (ROS) production, have been observed in both humans and AD animal models [12, 13, 14].

The mitogen‐activated protein kinase (MAPK) pathway, consisting of the Ras–Raf–MEK–ERK signalling cascade, regulates genes that control cellular development, differentiation, proliferation and apoptosis [15, 16, 17]. The MAPK cascades modulate multiple key stress‐related responses, as evidenced by astrocyte activation during AD pathogenesis [18]. Ras signalling has been linked to AD by regulating induction and post‐translational modification of tau and amyloid‐beta precursor protein (APP) [19, 20]. In the frontal cortex of early‐stage AD, up‐regulation of Ras expression acts on lysosomes and glycohydrolases, contributing to Aβ deposition and the formation of paired helical filaments, which suggests progression of AD neurodegeneration [21, 22]. RAS exists in three different isoforms (K‐RAS, H‐RAS and N‐RAS) with high sequence homology [23]. Mitogen‐activated protein kinase kinase kinase 11 (MAP3K11), known as MLK3 or SPRK, belongs to the serine/threonine protein kinase family and is encoded by the MAP3K11 gene in humans [24]. MAP3K11 expression is up‐regulated in neurodegenerative diseases [25], and silencing MAP3K11 inhibits ferroptosis [26, 27]. The solute carrier family 3 member 2, referred to as SLC3A2, CD98hc or 4F2hc, is a type II membrane protein [28, 29]. As a key target for ferroptosis [30], SLC3A2 expression is reduced in AD, while increased SLC3A2 expression inhibits ferroptosis [31, 32]. In AD research, glutathione peroxidase 4 (GPX4), an enzyme that helps neutralize lipid peroxides and supports the cellular antioxidant defence, has attracted attention due to its role in regulating ferroptosis [33]. For AD, a lack of GPX4 exacerbates neuronal death via ferroptosis, whereas an elevated level of GPX4 offers defence by diminishing lipid peroxidation and preserving neuronal integrity [34, 35]. Recent research has highlighted the importance of GPX4 activation in mitigating ferroptosis and AD pathology [9]. Ferroptosis inhibitors, such as deferoxamine [36], ferrostatin‐1 (Fer‐1) and liproxstatin‐1, have also been shown to enhance cognitive function in AD models [37, 38]. In light of these findings, inhibiting ferroptosis may prove to be a novel therapeutic approach for AD.

TARDBP (also known as TDP‐43) is involved in RNA splicing, stabilization and gene expression regulation [39]. Dysfunction of TARDBP is implicated in various neurodegenerative diseases [40, 41, 42]. An estimated 30%–70% of AD patients suffer from TDP‐43 proteinopathy [43]. Under normal conditions, TDP‐43 is mainly nuclear. In disease, TDP‐43 is cleared from the nucleus and accumulates in phosphorylated and poly‐ubiquitinated cytoplasmic inclusions [44, 45, 46]. The present study aimed to investigate the potential mechanisms by which TARDBP contributes to AD pathology by promoting ferroptosis.

## 2 Materials and Methods

### 2.1 AD Rat Model Establishment

Adult male SD rats (12–16 weeks; 280–300 g; Vital River Co. Ltd., Beijing, China) were raised under standard conditions (temperature, 22°C ± 2°C; light and dark cycle, 12–12 h) and had ad libitum access to food and water. An AD rat model was established by injecting homocysteine (Hcy) as previously described [47]. Briefly, Hcy was prepared in a 0.9% saline to a concentration of 400 μg/mL and administered to rats via tail vein injection at a dose of 400 μg/kg daily for 14 days. The sham‐operated group received equivalent saline injections. All procedures were approved by the Ethics Committee of Xuzhou Medical University (Approval number: 20240228).

### 2.2 Animal Groups

Recombinant adeno‐associated virus type 2 (rAAV) encoding TARDBP short hairpin RNA (sh‐TARDBP), a negative control shRNA (sh‐NC), a KRAS overexpression construct (oe‐KRAS) or its negative control (oe‐NC) were designed and synthesized by Vigene Bioscience (Shanghai, China). Ferrostatin‐1 (Fer‐1; S7243, Selleck Chemicals, USA) was diluted with 0.01% dimethyl sulfoxide (DMSO) saline. A total of seven groups of SD rats were randomly assigned: Sham group (no AD modelling); AD group (AD modelling); Fer‐1 group (after the 14‐day Hcy induction period, a single dose of 10 mg/kg Fer‐1 was administered by tail vein injection); sh‐NC group (after AD induction, 1 × 10^11^ vg/mL rAAV‐sh‐NC was injected into the tail vein); sh‐TARDBP group (after AD induction, 1 × 10^11^ vg/mL rAAV‐sh‐TARDBP was injected into the tail vein); sh‐TARDBP + oe‐NC group (after AD induction, a mixture of 1 × 10^11^ vg/mL rAAV‐sh‐TARDBP and oe‐NC was injected into the tail vein); sh‐TARDBP + oe‐KRAS group (after AD induction, a mixture of 1 × 10^11^ vg/mL rAAV‐sh‐TARDBP and oe‐KRAS was injected into the tail vein). Virus injections were administered after the 14‐day Hcy induction, representing a therapeutic intervention paradigm.

### 2.3 Morris Water Maze (MWM) Test

The MWM test was performed with minor modifications to a previously described protocol [48]. The MWM apparatus (XR‐XM101; Shanghai Xinruan, China) consisted of a circular pool (150 cm in diameter, 60 cm high) filled with tap water (30 cm deep, 25°C). The pool was divided into four quadrants of equal area. A circular platform (12 cm in diameter) was positioned 3 cm below the water surface in one of the quadrants. Rats were trained for five consecutive days, with four trials per day. Each acquisition trial consisted of placing a rat in a randomly selected quadrant and allowing it 60 s to locate the platform and 10 s to remain on it. Rats that failed to locate the platform within 60 s were manually placed on the platform and were allowed to stay there for 10 s. The escape latency was recorded. After the acquisition phase, the platform was removed, and a probe trial was conducted to assess the time spent in the target quadrant and the number of platform crossings.

### 2.4 Contextual Fear Test

Contextual fear test was conducted as described previously [47]. Rats were placed in a test chamber (32 × 26 × 26 cm) for 3 min for habituation. They were then subjected to two foot shocks (0.5 mA, 2 s duration) at 2‐min intervals, and then returned to their home cages. After 24 h, the rats were placed back into the same chamber without any electric shock to evaluate fear memory. Freezing behaviour, defined as the absence of all movement except respiration, was recorded, and the total freezing time was measured.

### 2.5 Haematoxylin and Eosin (HE) Staining

Rats were euthanized by intravenous injection of pentobarbital sodium (100 mg/kg). Rat brains were perfused with 4% paraformaldehyde (Sigma‐Aldrich, MO, USA), and then the hippocampus was dissected and fixed in 4% paraformaldehyde at 4°C for 12 h. On the following day, the tissues were embedded and sectioned into 4‐μm‐thick sections for HE staining. In brief, the sections were heated at 60°C overnight, successively dewaxed in xylene I and II for 20 min each, and incubated in a graded ethanol series (100%, 100%, 95%, 80% and 70%) for 5 min per solution. The dewaxed sections were stained with haematoxylin (Sigma‐Aldrich) for 10 min and eosin (Sigma‐Aldrich) for 30 s, followed by dehydration and mounting [49].

### 2.6 Perls' Blue Staining

Intracellular iron deposition in the hippocampus of AD rats was detected by Perls' blue staining. The hippocampal slices were stained with potassium ferrocyanide and nuclear fast red following the manufacturer's instructions (Solarbio, Beijing, China). Stained sections were observed under a Leica microscope (DM4B, Wetzlar, Germany) to assess morphological changes and iron distribution.

### 2.7 Cell Model Establishment

PC12 cells (Shanghai Institute of Cell Research, Shanghai, China) were cultured in DMEM (Gibco, NY, USA) containing 10% foetal bovine serum and 1% penicillin/streptomycin (Sigma‐Aldrich) at 37°C in an atmosphere of 5% CO_2_. Aβ_1–42_ was purchased from Sigma‐Aldrich. Aβ_1–42_ oligomers were prepared according to a previously described method [50]. Briefly, Aβ_1–42_ was initially dissolved in ice‐cold hexafluoroisopropanol (HFIP) to a concentration of 1 mmol/L. After sonication, incubation at room temperature and lyophilization, the resulting Aβ_1–42_ peptide film was re‐dissolved in DMSO. Serum‐free DMEM was then added, and the mixture was incubated overnight at 4°C to allow oligomer formation. PC12 cells were treated with 20 μM Aβ_1–42_ oligomers to establish an AD cell model [51].

### 2.8 Subcellular Fractionation Analysis

To determine the cellular localization of TARDBP, nuclear and cytoplasmic fractions were isolated using the NUCLEI EZ PREP Isolation Kit (Sigma‐Aldrich) according to the manufacturer's instructions. Western blot was then performed to detect TARDBP protein expression in the nuclear and cytoplasmic fractions, with histone H3 and GAPDH serving as loading controls for the nuclear and cytoplasmic fractions, respectively.

### 2.9 RNA Immunoprecipitation (RIP) Assay

An RIP assay was performed to investigate the interaction between TARDBP protein and KRAS mRNA using the Magna RIP RNA‐Binding Protein Immunoprecipitation Kit (Sigma‐Aldrich). PC12 cells were first lysed using RIP‐compatible lysis buffer. Anti‐TARDBP antibody (1:1000; R&D Systems, MN, USA) or normal rabbit IgG was conjugated to protein A/G magnetic beads. The antibody‐coated beads were then incubated with the cell lysate. Following immunoprecipitation, bound RNA was extracted and subjected to quantitative analysis by RT‐qPCR.

### 2.10 Determination of Actinomycin D

To assess mRNA stability, PC12 cells were treated with actinomycin D (5 μg/mL) and collected at the indicated time points (0, 4, 8 and 12 h) for KRAS mRNA measurement by RT‐qPCR.

### 2.11 Cell Transfection

Cells were divided into seven groups: control group (without any treatment), Aβ_1–42_ group (treated with 20 μM Aβ_1–42_), Fer‐1 group (treated with 10 μM Aβ_1–42_ and 2 μM Fer‐1), sh‐NC group (treated with Aβ_1–42_ and transfected with sh‐NC), sh‐TARDBP group (treated with Aβ_1–42_ and transfected with sh‐TARDBP), sh‐TARDBP + oe‐NC group (treated with Aβ_1–42_ and transfected with sh‐TARDBP + oe‐NC), sh‐TARDBP + oe‐KRAS group (treated with Aβ_1–42_ and transfected with sh‐TARDBP + oe‐KRAS). Transfections were performed according to the manufacturer's instructions for the Lipofectamine 2000 kit (Thermo Fisher Scientific, MA, USA).

### 2.12 MTT Test

PC12 cells were cultivated in 96‐well plates (3 × 10^4^/well) overnight and then incubated with MTT solution (10 μL, 5 mg/mL) at 37°C for 4 h. After removing the medium, 100 μL of DMSO was added, and absorbance was measured at 570 nm using a Bio‐Tek Instruments microplate reader.

### 2.13 Determination of Lactate Dehydrogenase (LDH) Content

LDH levels were measured using the LDH assay kit (CK12, Dojindo, Japan). The absorbance at 490 nm was recorded.

### 2.14 Determination of ROS

Cells were incubated with 10 μM 2′,7′‐dichlorofluorescein diacetate (S0033M, Beyotime, Shanghai, China) at 37°C for 20 min. Fluorescence (excitation at 488 nm) was observed using a fluorescence microscope.

### 2.15 Determination of Fe^2+^ Concentration

The Fe^2+^ concentration in cell lysates was determined using a Ferrous Iron Colorimetric Assay Kit (E‐BC‐K773‐M, Elabscience, Wuhan, China) and the absorbance was measured at 590 nm using a microplate reader.

### 2.16 Detection of Lipid Peroxidation Levels

Lipid peroxidation levels in PC12 cells were evaluated using the fluorescent probe C11‐BODIPY 581/591. Briefly, cells were rinsed three times with PBS and then incubated with culture medium containing 5 μM C11‐BODIPY 581/591 probe for 30 min at 37°C. After three additional PBS washes to eliminate unbound probe, fluorescence imaging was performed under a fluorescence microscope at excitation and emission wavelengths of 510 and 590 nm, respectively, following the manufacturer's instructions.

Malondialdehyde (MDA; D799761; Sangon Biotech Co. Ltd.) and 4‐hydroxynonenal (4‐HNE; D751041; Sangon Biotech Co. Ltd.) levels were quantified using the corresponding commercial assay kits according to the manufacturers' instructions. Optical density was measured using a microplate reader.

### 2.17 RT‐qPCR

Using TRIzol reagent (Invitrogen, CA, USA), total RNA was isolated. Then, RNA was reverse transcribed using the Reverse Transcription Kit (Toyobo, Japan), and RT‐qPCR was performed using SYBR Green PCR Master Mix (Takara, Japan) on a Roche real‐time PCR system (Roche, Basel, Switzerland) with the following thermal cycling conditions: initial denaturation at 95°C for 2 min, denaturation at 94°C for 20 s, annealing at 58°C for 20 s and extension at 72°C for 40 s, for 40 cycles. Relative quantification of target genes was performed using the 2^−ΔΔCt^ method. Table 1 displays the sequences of the primers.

**TABLE 1:** Primers.

Genes	Sequences (5′–3′)
TARDBP	Forward: TGGGAATCAGGGTGGATTTG
	Reverse: CCCAGCCAGAAGACTTAGAATC
KRAS	Forward: GGACTGGGGAGGGCTTTCT
	Reverse: GCCTGTTTTGTGTCTACTGTTCT
GAPDH	Forward: GTCGGTGTGAACGGATTTG
	Reverse: TCCCATTCTCAGCCTTGAC

Abbreviations: GAPDH, glyceraldehyde 3‐phosphate dehydrogenase; KRAS, Kirsten rat sarcoma viral oncogene homologue; TARDBP, transactive response DNA binding protein.

### 2.18 Western Blot

Total protein was extracted using RIPA buffer (Thermo Fisher Scientific). Protein concentrations were determined using a BCA kit (Thermo Fisher Scientific). Subsequently, the protein samples were separated by 10% SDS‐PAGE and transferred to polyvinylidene fluoride membranes. After blocking with 5% skim milk, the membranes were incubated overnight at 4°C with primary antibodies against TARDBP (1:2500, R&D Systems), phosphorylated TDP‐43 (p‐TDP‐43; 1:1000, Proteintech), KRAS (1:1000, Abcam), ERK (1:1000, Proteintech), p‐ERK (1:1000, Proteintech), MAP3K11 (1:1000, Cell Signaling Technology), SLC3A2 (1:500, Abcam), GPX4 (1:1000, Abcam) and GAPDH (1:1000, Abcam). The membranes were then incubated with horseradish peroxidase‐conjugated secondary antibody (1:5000, Abcam) for 2 h at 37°C, after which protein bands were visualized using enhanced chemiluminescence (Millipore, USA).

### 2.19 Statistical Analysis

All continuous data were analysed using SPSS 20.0 (IBM, NY, USA) and are presented as mean ± standard deviation (SD). For two‐group comparisons, intergroup differences were analysed using independent‐samples Student's *t*‐test, and for multiple group comparisons, one‐way analysis of variance (ANOVA) followed by Tukey's post hoc test was used. *p* < 0.05 was considered statistically significant.

## 3 Results

### 3.1 Fer‐1 Improves Memory Ability and Reduces Intracellular Iron Deposition in AD Rats

**FIGURE 1:**
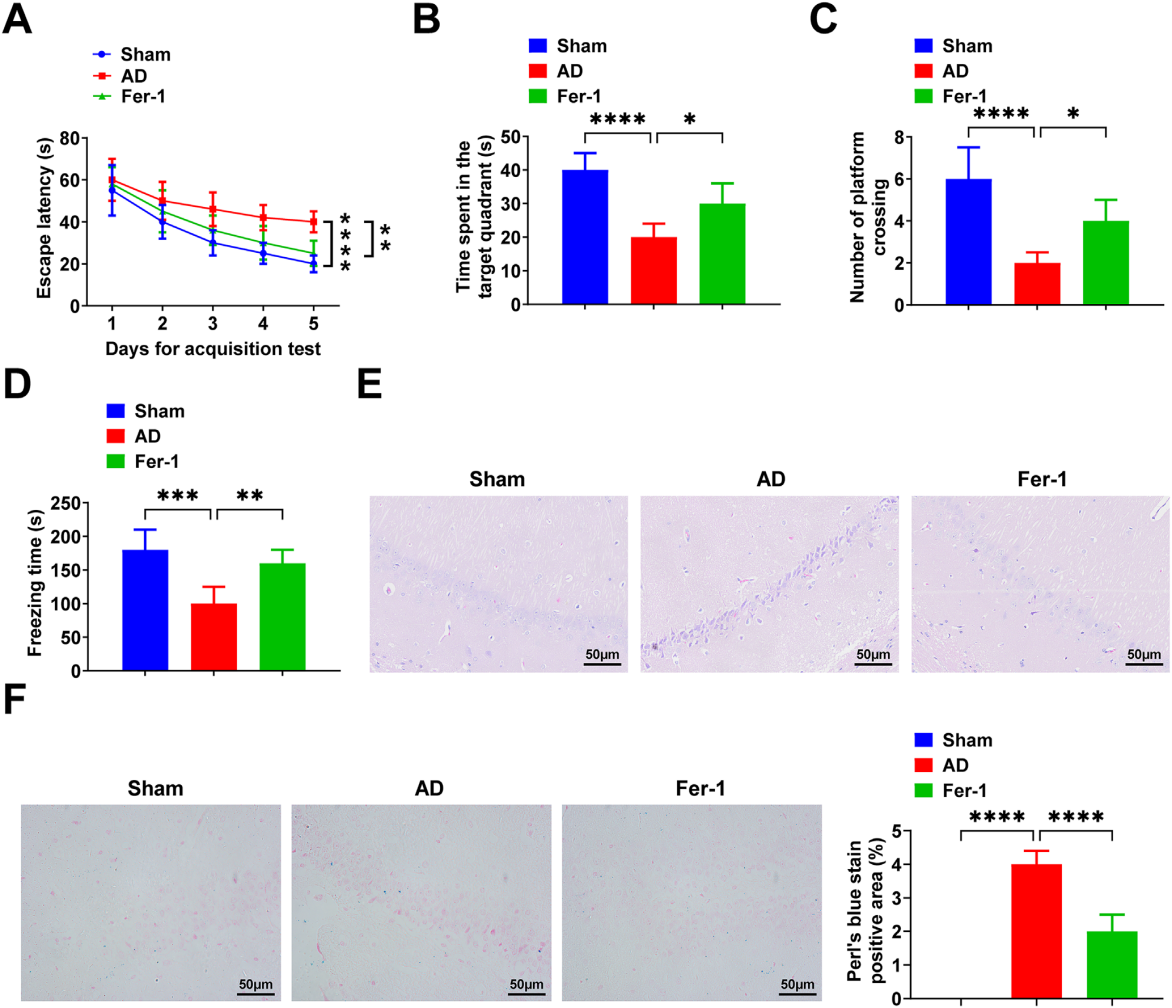
Fer‐1, a ferroptosis inhibitor, improves memory capacity and reduces intracellular iron deposition in the hippocampus of AD rats. (A–C) MWM test; (D) Contextual fear test; (E) HE staining of hippocampus CA1 region; (F) Perls' blue staining of hippocampus CA1 region. Data are expressed as mean ± standard deviation. *p < 0.05, **p < 0.01, ***p < 0.001, ****p < 0.0001.

SD rats were injected with Hcy daily for 14 consecutive days to simulate AD in vivo. The MWM test showed that AD rats had a longer escape latency, spent less time in the target quadrant and crossed the platform fewer times, whereas Fer‐1 decreased the escape latency and increased the stay in the target quadrant and the number of platforms traversed (Figure 1A–C). Contextual fear test results showed that AD rats had reduced total freezing time, whereas Fer‐1 increased total freezing time (Figure 1D). The morphology of hippocampal neurons was observed by HE staining, and the results showed that the hippocampal neuronal layer was disorganized with neuronal consolidation and cytoplasmic reduction in AD rats, whereas Fer‐1 ameliorated hippocampal neuronal pathological changes (Figure 1E). Observation of intracellular iron deposition in the hippocampus of AD rats by Perls' blue staining showed that intracellular iron deposition was increased in AD rats, whereas Fer‐1 reduced intracellular iron deposition (Figure 1F). Additionally, it was observed that with prolonged Hcy injection time, the protein expression levels of TARDBP, KRAS and MAP3K11 increased, while those of SLC3A2 and GPX4 decreased (Figure S1A).

### 3.2 Down‐Regulating TARDBP Improves Memory Ability and Reduces Intracellular Iron Deposition in AD Rats

**FIGURE 2:**
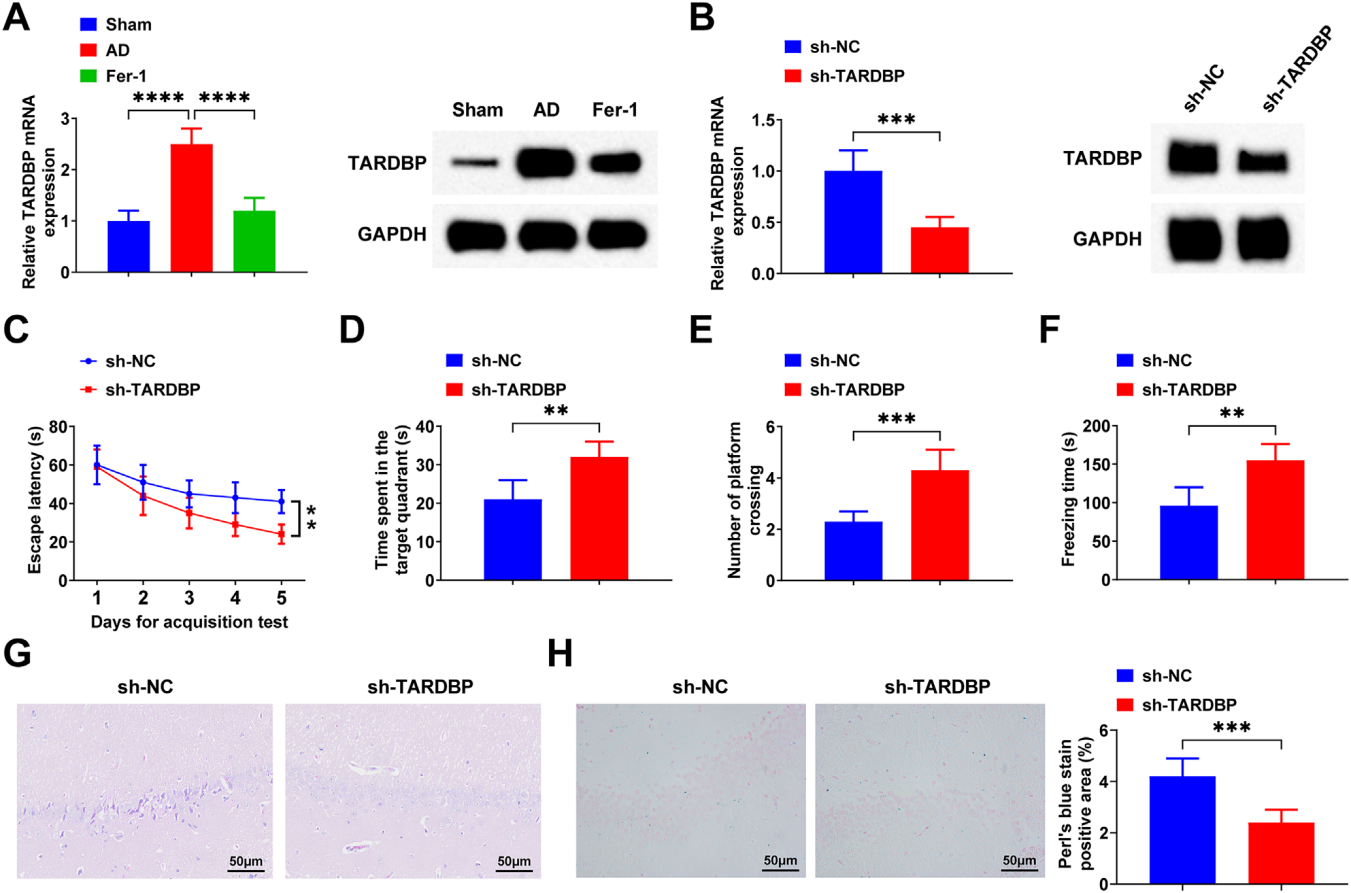
Down‐regulation of TARDBP improves memory capacity and reduces intracellular iron deposition in the hippocampus of AD rats. (A, B) RT‐qPCR and Western blot to detect TARDBP expression; (C–E) MWM test; (F) Contextual fear test; (G) HE staining of hippocampus CA1 region; (H) Perls' blue staining of hippocampus CA1 region. Data are expressed as mean ± standard deviation. *p < 0.05, **p < 0.01, ***p < 0.001, ****p < 0.0001.

RT‐qPCR results showed that the mRNA expression level of TARDBP was significantly up‐regulated in AD rats compared to the Sham group, reaching 2.5‐fold of the Sham level (Figure 2A). Subcellular fractionation was performed to analyse the subcellular localization of TARDBP protein. The results showed that in the brain tissue of AD rats, TARDBP protein expression was significantly decreased in the nucleus and increased in the cytoplasm (Figure S1B). Moreover, the level of phosphorylated TDP‐43 was elevated in the brain tissue of AD rats (Figure S1C). After Fer‐1 treatment, TARDBP mRNA expression decreased to 0.48‐fold of that in the AD group (Figure 2A). Western blot analysis further indicated that TARDBP protein expression was increased in the AD group, and Fer‐1 treatment suppressed its expression (Figure 2A). Based on these findings, the role of TARDBP in AD rats was further investigated. Following tail‐vein injection of sh‐NC or sh‐TARDBP adenovirus into AD rats, RT‐qPCR confirmed that the TARDBP mRNA level in the sh‐TARDBP group was reduced to 0.45‐fold of that in the sh‐NC group (Figure 2B). Western blot results also showed decreased TARDBP protein expression after sh‐TARDBP adenovirus injection (Figure 2B). The MWM test showed that down‐regulating TARDBP reduced the escape latency and increased the time spent in the target quadrant and the number of times of crossing the platform (Figure 2C–E). Also, down‐regulating TARDBP increased the total freezing time (Figure 2F). Down‐regulating TARDBP improved the pathological changes of hippocampal neurons (Figure 2G) and decreased intracellular iron deposition (Figure 2H).

### 3.3 Down‐Regulation of TARDBP Attenuates KRAS mRNA Stability

**FIGURE 3:**
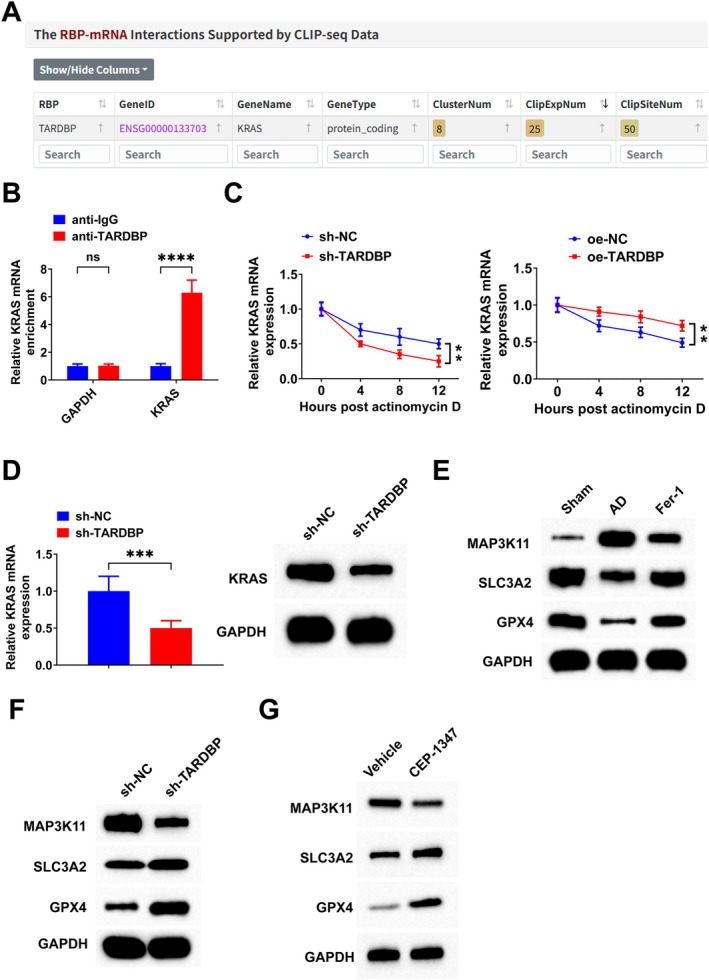
Down‐regulation of TARDBP attenuates the mRNA stability of KRAS. (A) Bioinformatic analysis via the ENCORI website (https://rnasysu.com/encori/) predicted an interaction between TARDBP and KRAS mRNA; (B) RIP assay confirmed the interaction between TARDBP and KRAS mRNA; (C) Actinomycin D assay; (D) RT‐qPCR and Western blot to detect KRAS expression; (E–G) Western blot to detect MAP3K11, SLC3A2 and GPX4 protein expression. Data are expressed as mean ± standard deviation. *p < 0.05, **p < 0.01, ***p < 0.001, ****p < 0.0001.

Next, the downstream regulatory mechanisms by which TARDBP participates in ferroptosis were explored. It has been shown that the RAS/MAPK signalling pathway is involved in the regulation of ferroptosis [52]. Bioinformatic analysis using the ENCORI database (https://rnasysu.com/encori/) revealed a potential interaction between TARDBP and KRAS mRNA (Figure 3A), which was subsequently validated by an RIP assay (Figure 3B). The mRNA stability of KRAS was assessed by an actinomycin D assay, which showed that down‐regulating TARDBP attenuated the mRNA stability of KRAS, while up‐regulating TARDBP enhanced it (Figure 3C). RT‐qPCR results showed that down‐regulation of TARDBP significantly decreased KRAS mRNA levels; compared with the sh‐NC group, KRAS mRNA expression in the sh‐TARDBP group was reduced to 0.5‐fold (Figure 3D). Western blot analysis further indicated that down‐regulating TARDBP inhibited KRAS protein expression (Figure 3D). The protein expression levels of ERK and MAP3K11 (key regulators of the MAPK signalling pathway), as well as SLC3A2 and GPX4 (known inhibitors of ferroptosis), were examined by Western blot. In AD rats, p‐ERK and MAP3K11 expression was elevated, while SLC3A2 and GPX4 were reduced. In contrast, Fer‐1 treatment inhibited MAP3K11 and promoted the protein expression of SLC3A2 and GPX4 (Figure 3E and Figure S2A). Moreover, down‐regulating TARDBP decreased p‐ERK and MAP3K11 protein expression and elevated SLC3A2 and GPX4 protein expression (Figure 3F and Figure S2B). Furthermore, following treatment with the MAP3K11 inhibitor CEP‐1347 (1 μM; Tocris, Bristol, UK), MAP3K11 expression was reduced, while SLC3A2 and GPX4 expression was increased (Figure 3G).

### 3.4 Up‐Regulating KRAS Attenuates the Ameliorative Effect of Down‐Regulation of TARDBP in AD Rats

**FIGURE 4:**
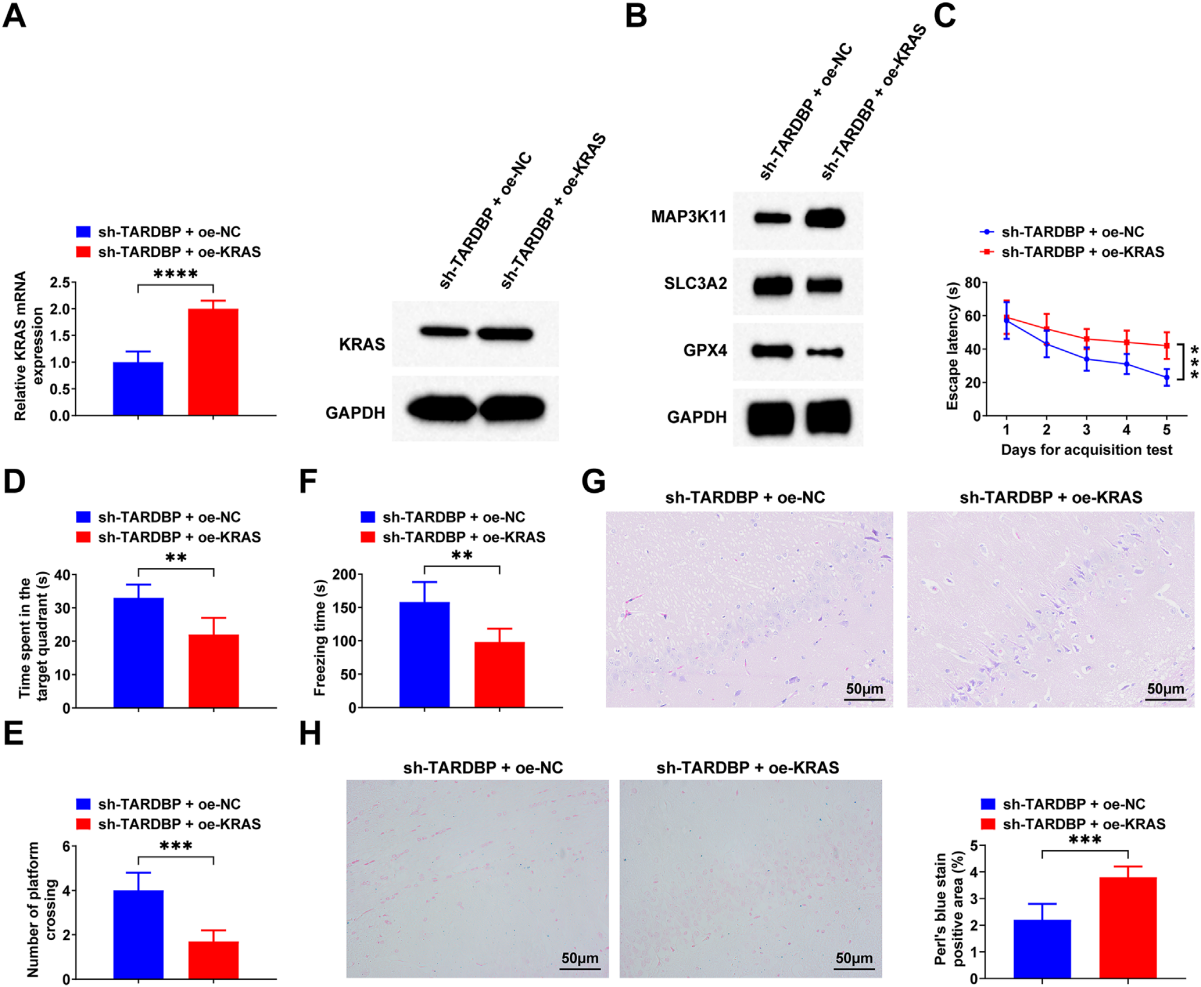
Up‐regulating KRAS attenuates the ameliorative effect of down‐regulation of TARDBP in AD rats. (A) RT‐qPCR and Western blot for KRAS expression; (B) Western blot for MAP3K11, SLC3A2 and GPX4 protein expression; (C–E) MWM test; (F) Contextual fear test; (G) HE staining of hippocampus CA1 region; (H) Perls' blue staining of hippocampus CA1 region. Data are expressed as mean ± standard deviation. *p < 0.05, **p < 0.01, ***p < 0.001, ****p < 0.0001.

The sh‐TARDBP + oe‐NC or sh‐TARDBP + oe‐KRAS adenovirus was injected into AD rats, and the success of the injection was verified by RT‐qPCR and Western blot (Figure 4A). The Western blot results showed that after up‐regulating KRAS, p‐ERK and MAP3K11 protein levels increased, and SLC3A2 and GPX4 protein expression decreased (Figure 4B and Figure S2C). After up‐regulation of KRAS, the escape latency increased, and the time spent in the target quadrant and the number of crossing platforms decreased (Figure 4C–E). Contextual fear test results showed that total freezing time decreased after up‐regulation of KRAS (Figure 4F). HE staining showed that hippocampal neuronal pathology worsened after up‐regulating KRAS (Figure 4G). Perls' blue staining results showed that intracellular iron deposition increased after up‐regulating KRAS (Figure 4H).

### 3.5 Fer‐1 Inhibits Aβ_1–42_‐Induced PC12 Cell Damage, ROS Production, Iron Accumulation and Lipid Peroxidation

**FIGURE 5:**
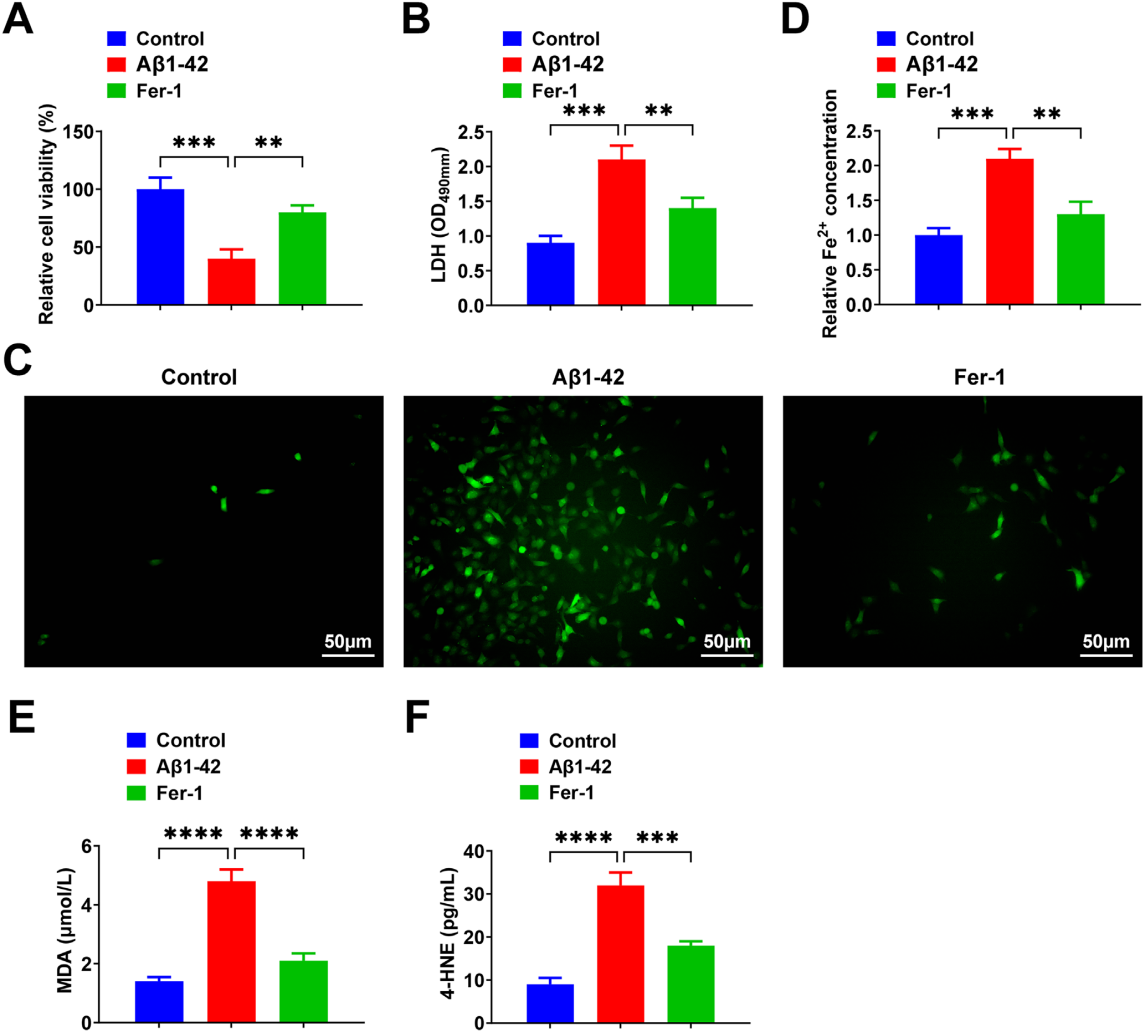
Fer‐1 inhibits Aβ_1–42_‐induced PC12 cell injury, ROS production and iron accumulation. (A) Cell viability detected by MTT; (B) LDH content detected by LDH assay kit; (C) Intracellular ROS assay; (D) Fe^2+^ concentration assay; (E, F) Levels of MDA and 4‐HNE were measured using commercial kits. Data are expressed as mean ± standard deviation. *p < 0.05, **p < 0.01, ***p < 0.001, ****p < 0.0001.

Then, we verified our conclusions in in vitro experiments. PC12 cells were treated with 20 μM Aβ_1–42_ to establish an AD cell model. Subcellular fractionation analysis was performed to examine TARDBP localization. The results showed that after Aβ_1–42_ treatment, TARDBP protein expression decreased in the nucleus and increased in the cytoplasm (Figure S1D). Moreover, the level of phosphorylated TDP‐43 was elevated in AD rat brain tissue (Figure S1E). Lipid peroxidation levels in PC12 cells were detected using the C11 BODIPY probe, and the results showed that lipid peroxidation levels were increased after Aβ_1–42_ treatment (Figure S1F). Cell viability was detected by MTT, indicating that Aβ_1–42_ inhibited cell viability, while Fer‐1 attenuated the inhibitory effect of Aβ_1–42_ on cell viability (Figure 5A). Detection of LDH content showed that Aβ_1–42_ promoted LDH release, whereas Fer‐1 attenuated this effect (Figure 5B). Aβ_1–42_ promoted intracellular ROS production and iron accumulation, whereas Fer‐1 significantly suppressed these Aβ_1–42_‐induced increases (Figure 5C,D). Furthermore, after Aβ_1–42_ treatment, MDA and 4‐HNE levels in PC12 cells were increased, while Fer‐1 decreased these levels (Figure 5E,F). Additionally, it was observed that liproxstatin‐1 (Lip‐1; 0.5 μM; Selleck Chemicals, USA), another specific ferroptosis inhibitor, also suppressed Aβ_1–42_‐induced PC12 cell damage, ROS production, iron accumulation and lipid peroxidation (Figure S3A–E).

### 3.6 Up‐Regulating KRAS Attenuates the Ameliorative Effect of Down‐Regulation of TARDBP on Aβ_1–42_‐Induced PC12 Cells

**FIGURE 6:**
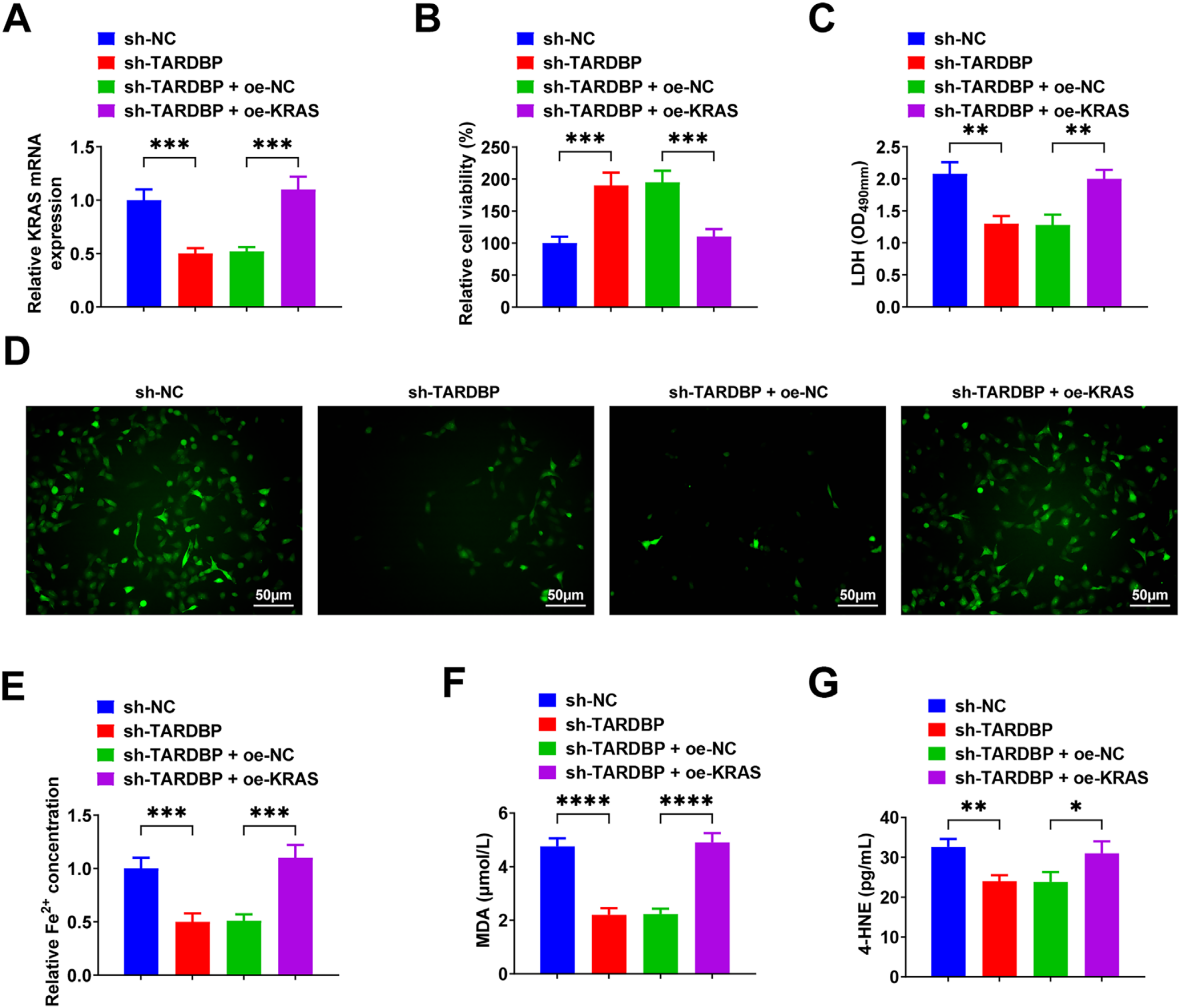
Up‐regulation of KRAS attenuates the ameliorative effect of down‐regulation of TARDBP on Aβ_1–42_‐induced PC12 cells. (A) RT‐qPCR to verify successful transfection; (B) MTT to detect cell viability; (C) LDH assay kit to detect LDH content; (D) Intracellular ROS assay; (E) Fe^2+^ concentration assay; (F, G) Levels of MDA and 4‐HNE were measured using commercial kits. Data are expressed as mean ± standard deviation. *p < 0.05, **p < 0.01, ***p < 0.001, ****p < 0.0001.

sh‐NC, sh‐TARDBP, sh‐TARDBP + oe‐NC and sh‐TARDBP + oe‐KRAS were transfected into Aβ_1–42_‐treated PC12 cells, and transfection efficiency was verified by RT‐qPCR (Figure 6A). Up‐regulating KRAS attenuated the inhibitory effects of TARDBP knockdown on Aβ_1–42_‐induced PC12 cell injury, ROS production, iron accumulation and lipid peroxidation (Figure 6B–G).

## 4 Discussion

Cognitive impairment and memory decline are well established AD symptoms, which have been highly correlated with accumulation of Aβ plaques, neuronal apoptosis and neurofibrillary tangles [53]. Essentially, Aβ leads to the shrinkage of synaptic spines, diminishes the strength of synaptic transmission and eventually interferes with long‐term potentiation, a dominant form of synaptic plasticity that may facilitate learning, cognition, and memory processes in the brain [53, 54, 55, 56, 57]. PC12 cells have been frequently used to establish neuronal cell models for studying the functions and mechanisms of molecules [47, 51, 58]. Previous studies have used Hcy‐induced rats for in vivo experimental studies [59, 60]. AD has been linked to hyperhomocysteinemia as an independent risk factor [61, 62]. As a contributing factor to neuronal pathology, hyperhomocysteinemia damages neuronal membrane components by disrupting the oxidative state in brain parenchyma [63]. Hcy acts as a biomarker of AD dementia, as well as an indicator of cognitive impairment [64]. In this study, Aβ_1–42_‐treated PC12 cells were used to establish a cellular model, and Hcy was used to induce an AD rat model.

Increasing evidence emphasizes the critical role of iron oxidation in neuronal death and neuroinflammation [65, 66, 67]. Lipid peroxidation is increased in brain tissue and cerebrospinal fluid of patients with neurological disorders [68]. Iron accumulation in the brain is strongly associated with cognitive decline and disease progression in patients with cognitive decline [69, 70]. In addition, treatment with iron chelators has been effective in AD clinical trials [71]. Inducers of ferroptosis markedly enhance neuronal death in lab conditions, linking this process to ROS overproduction. Nonetheless, the prevention of cell death is achievable using the iron oxidase inhibitors and ROS scavengers [72, 73]. GPX4 is an enzyme that uses glutathione to convert lipid peroxides to non‐toxic lipid alcohols, which prevents ferroptosis [74]. Neurodegenerative diseases can be improved by overexpressing GPX4, which inhibits lipid peroxide production and neuronal death [75, 76, 77, 78]. In the present study, Fer‐1, a ferroptosis inhibitor, improved memory capacity and reduced intracellular iron deposition in the hippocampus of AD rats; additionally, Fer‐1 inhibited Aβ_1–42_‐induced PC12 cell injury, ROS production and iron accumulation.

TARDBP is an RNA‐binding protein belonging to the heterogeneous ribonucleoprotein family (hnRNPs) [79]. Physiologically, TARDBP is located mainly in the nucleus, although it is also present in the cytoplasm, particularly in its aggregated form [80]. AD patients affected by TDP‐43 protein pathology exhibit greater disease severity as evidenced by poorer memory and more severe hippocampal atrophy than AD patients without TDP‐43 pathology [81, 82, 83]. TDP‐43 overproduction exacerbates AD pathology and synaptic and cognitive decline after traumatic brain injury [84]. In the present study, TARDBP expression was up‐regulated in AD rats, and down‐regulating TARDBP improved memory capacity and reduced intracellular iron deposition in the hippocampus of AD rats. In addition, down‐regulating TARDBP inhibited Aβ_1–42_‐induced PC12 cell injury, ROS production and iron accumulation.

Gene expression is regulated post‐transcriptionally by RNA‐binding proteins [85, 86, 87]. In this study, down‐regulating TARDBP attenuated the mRNA stability of KRAS and inhibited KRAS expression. Moreover, up‐regulating KRAS attenuated the ameliorative effects of TARDBP down‐regulation on AD rats and Aβ_1–42_‐induced PC12 cells. Emerging evidence has demonstrated that suppressing KRAS expression can inhibit ferroptosis in several disease models [88, 89, 90]. Furthermore, it has been reported that RAS expression is elevated in human AD brain tissue samples [91]. The RAS/MAPK signalling pathway is involved in the regulation of ferroptosis [52]. MAP3K11 is a key regulator of MAPK signalling [92], and SLC3A2 and GPX4 are ferroptosis inhibitors [93]. In this study, MAP3K11 protein expression was elevated and SLC3A2 and GPX4 protein expression were decreased in AD rats, and down‐regulating TARDBP inhibited MAP3K11 and promoted SLC3A2 and GPX4 protein expression.

However, there are limitations to this study. The expression of TARDBP in clinical samples remains unknown, since it was only detected in AD cells and animal models. Future studies will assess the therapeutic and diagnostic value of TARDBP in AD clinical samples. Furthermore, the specific molecular mechanism by which KRAS influences the MAP3K11/SLC3A2/GPX4 axis requires further clarification.

## 5 Conclusions

TARDBP promotes AD by enhancing the stability of KRAS mRNA through mediating the MAP3K11/SLC3A2/GPX4 axis. This finding could serve as a potential target for the treatment or diagnosis of AD.

## Author Contributions

**QiTao Zhao:** conceptualization (equal), writing – original draft (equal). **YaDong Yu:** conceptualization (equal), writing – original draft (equal). **FuJiang Wang:** conceptualization (equal), data curation (equal), formal analysis (equal). **Ya Wang:** conceptualization (equal), data curation (equal), formal analysis (equal). **Peng Shao:** conceptualization (equal), formal analysis (equal), writing – review and editing (equal). **LianDong Zhao:** conceptualization (equal), data curation (equal), writing – original draft (equal), writing – review and editing (equal).

## Funding

This work was supported by Huai'an Natural Science Research Program (No. HAB202113).

## Ethics Statement

All animal experiments were complied with the ARRIVE guidelines and performed in accordance with the National Institutes of Health Guide for the Care and Use of Laboratory Animals. The experiments were approved by the Institutional Animal Care and Use Committee of Xuzhou Medical University (approval number: 20240228).

## Conflicts of Interest

The authors declare no conflicts of interest.

## Supporting information


**Figure S1:** Regulation of TARDBP/TDP‐43 and lipid peroxidation in cellular and animal models of AD. (A) Western blot analysis of TARDBP, KRAS and MAP3K11/SLC3A2/GPX4 axis protein expression in rat brain tissue after Hcy injection for 3, 7 or 14 days; (B) Western blot analysis of TARDBP protein localization in AD rat brain tissue; (C) Western blot analysis of p‐TDP‐43 levels in AD rat brain tissue; (D) Western blot analysis of TARDBP protein localization in Aβ_1–42_‐treated PC12 cells; (E) Western blot analysis of p‐TDP‐43 levels in Aβ_1–42_‐treated PC12 cells; (F) Lipid peroxidation was detected using the C11 BODIPY probe after Aβ_1–42_ treatment.

**Figure S2:** p‐ERK expression in different treatment groups. (A–C) Western blot analysis of p‐ERK expression in AD rat brain tissue.

**Figure S3:** Lip‐1 inhibits Aβ_1–42_‐induced PC12 cell injury, ROS production, iron accumulation and lipid peroxidation. (A) Cell viability detected by MTT; (B) LDH content detected by LDH assay kit; (C) Intracellular ROS assay; (D) Fe^2+^ concentration assay; (E, F) Levels of MDA and 4‐HNE were measured using commercial kits. Data are expressed as mean ± standard deviation. *p < 0.05, **p < 0.01, ***p < 0.001, ****p < 0.0001.

## Data Availability

The datasets used and/or analysed during this study are available from the corresponding author on reasonable request.
