# Supplementary figures and images for: TARDBP Mediates the MAP3K11/SLC3A2/GPX4 Axis in Alzheimer's Disease Rats by Enhancing KRAS mRNA Stability
Source: J Cell Mol Med. 2026 May 17;30(10):e71181. doi: 10.1111/jcmm.71181 (PMC13180789; doi:10.1111/jcmm.71181)

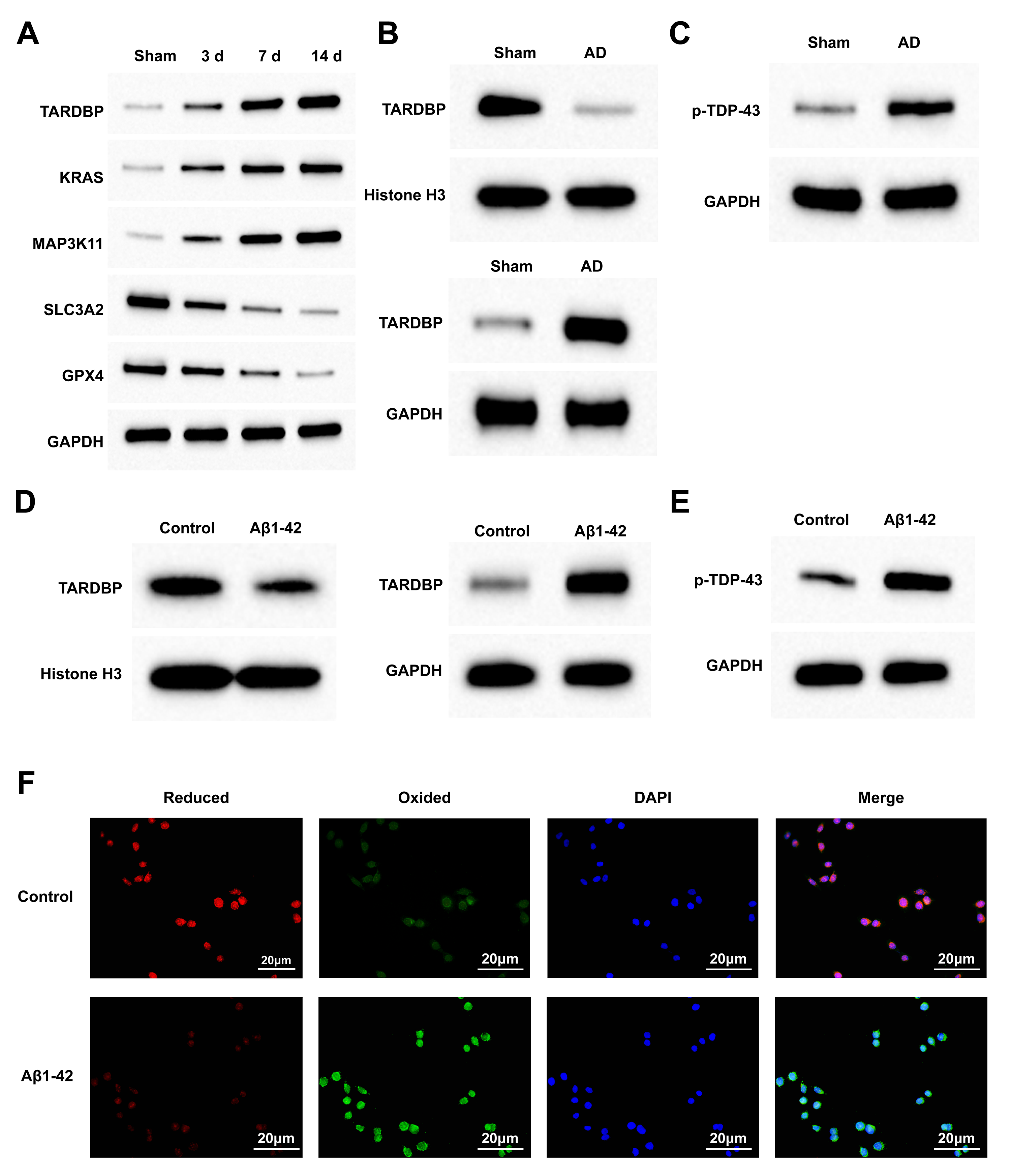

Supplement: Supplementary file 1 — Figure S1: Regulation of TARDBP/TDP‐43 and lipid peroxidation in cellular and animal models of AD. (A) Western blot analysis of TARDBP, KRAS and MAP3K11/SLC3A2/GPX4 axis protein expression in rat brain tissue after Hcy injection for 3, 7 or 14 days; (B) Western blot analysis of TARDBP protein localization in AD rat brain tissue; (C) Western blot analysis of p‐TDP‐43 levels in AD rat brain tissue; (D) Western blot analysis of TARDBP protein localization in Aβ1–42‐treated PC12 cells; (E) Western blot analysis of p‐TDP‐43 levels in Aβ1–42‐treated PC12 cells; (F) Lipid peroxidation was detected using the C11 BODIPY probe after Aβ1–42 treatment. [file JCMM-30-e71181-s001.tif]

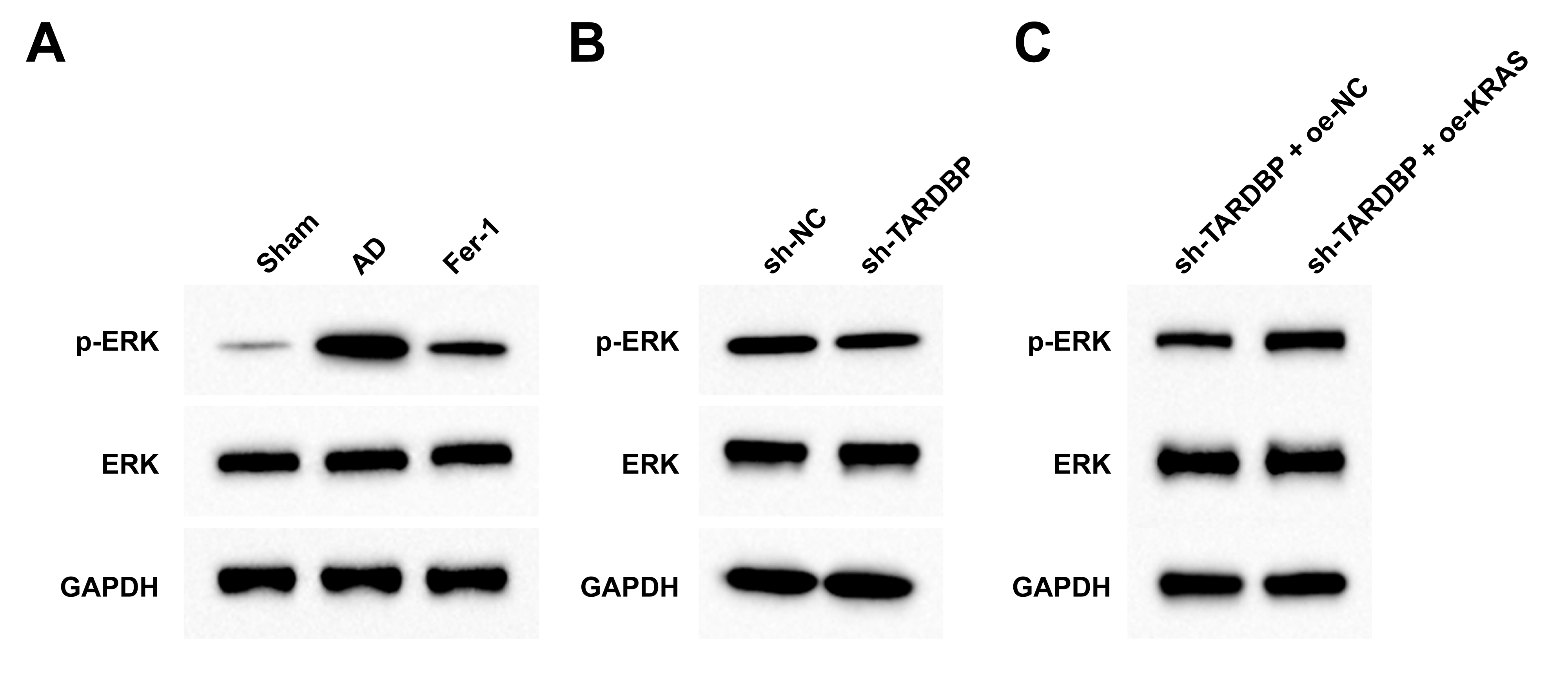

Supplement: Supplementary file 2 — Figure S2: p‐ERK expression in different treatment groups. (A–C) Western blot analysis of p‐ERK expression in AD rat brain tissue. [file JCMM-30-e71181-s002.tif]

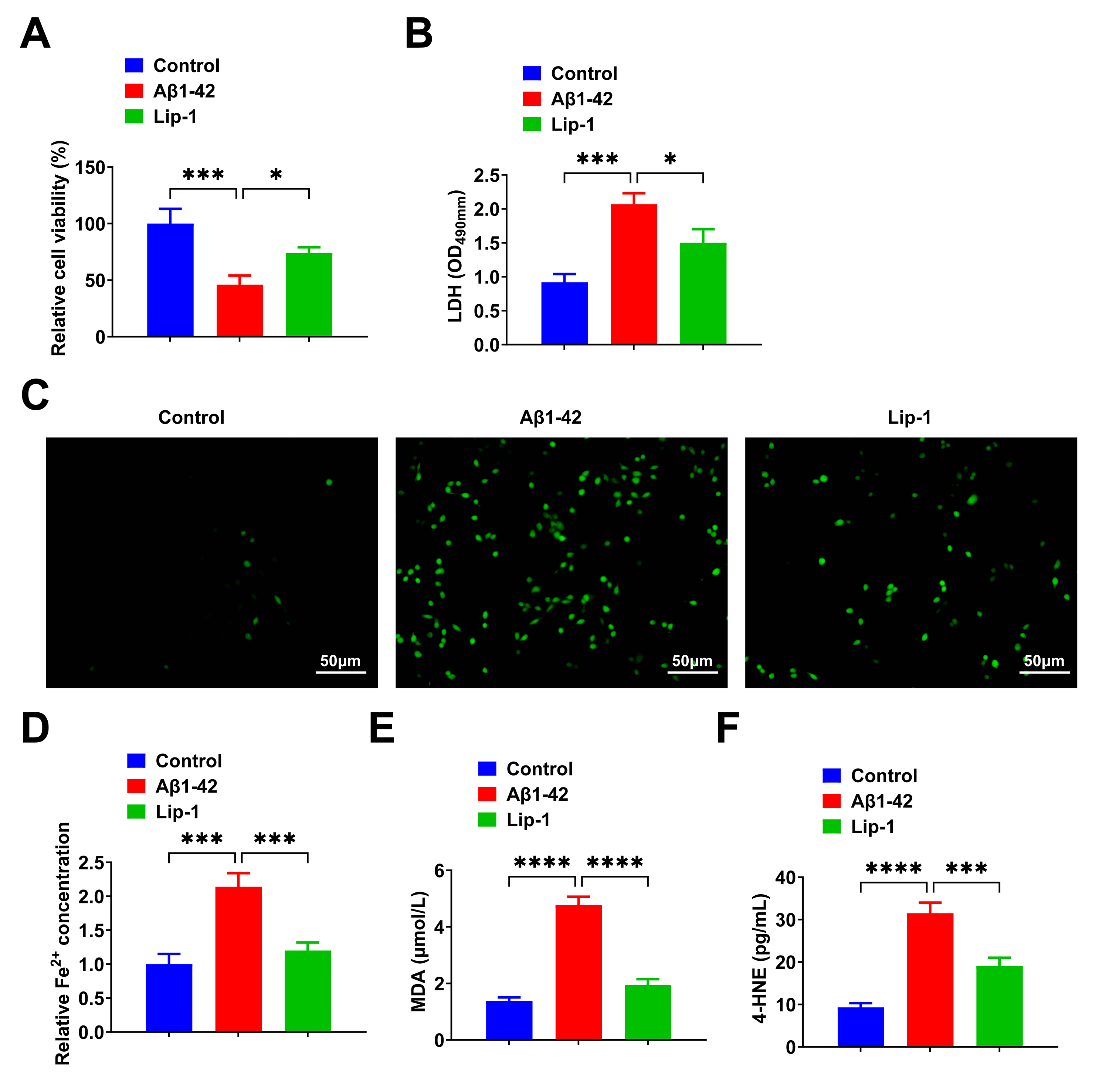

Supplement: Supplementary file 3 — Figure S3: Lip‐1 inhibits Aβ1–42‐induced PC12 cell injury, ROS production, iron accumulation and lipid peroxidation. (A) Cell viability detected by MTT; (B) LDH content detected by LDH assay kit; (C) Intracellular ROS assay; (D) Fe2+ concentration assay; (E, F) Levels of MDA and 4‐HNE were measured using commercial kits. Data are expressed as mean ± standard deviation. *p < 0.05, **p < 0.01, ***p < 0.001, ****p < 0.0001. [file JCMM-30-e71181-s003.tif]
